# Bis[1,3-bis­(diphenyl­phosphino)propane-κ^2^ *P*:*P*′]silver(I) bis­(chloro­difluoro­acetato-κ*O*)triphenyl­stannate(IV)

**DOI:** 10.1107/S1600536808010763

**Published:** 2008-04-23

**Authors:** Yin Yin Teo, Kong Mun Lo, Seik Weng Ng

**Affiliations:** a Department of Chemistry, University of Malaya, 50603 Kuala Lumpur, Malaysia

## Abstract

In the title salt, [Ag(C_27_H_26_P_2_)_2_][Sn(C_6_H_5_)_3_(C_2_ClF_2_O_2_)], the Ag^I^ atom exists in a tetra­hedral coordination geometry formed by four P atoms [Ag—P = 2.460 (1)–2.501 (1) Å], whereas the Sn^IV^ atom exists in a *trans*-trigonal–bipyramidal coordination geometry formed by two O [Sn—O = 2.208 (3) and 2.233 (3) Å] and three C atoms [Sn—C = 2.115 (4)–2.128 (4) Å;(Σ C—Sn—C)= 360.0 (6)°].

## Related literature

For the crystal structures of other bis­(chloro­difluoro­acetato)triorganostannates, see: Ng & Hook (1999 ▶); Teo *et al.* (2004 ▶, 2007 ▶, 2008 ▶). The structural chemistry of organotin carboxyl­ates has been reviewed by Tiekink (1991 ▶, 1994 ▶).
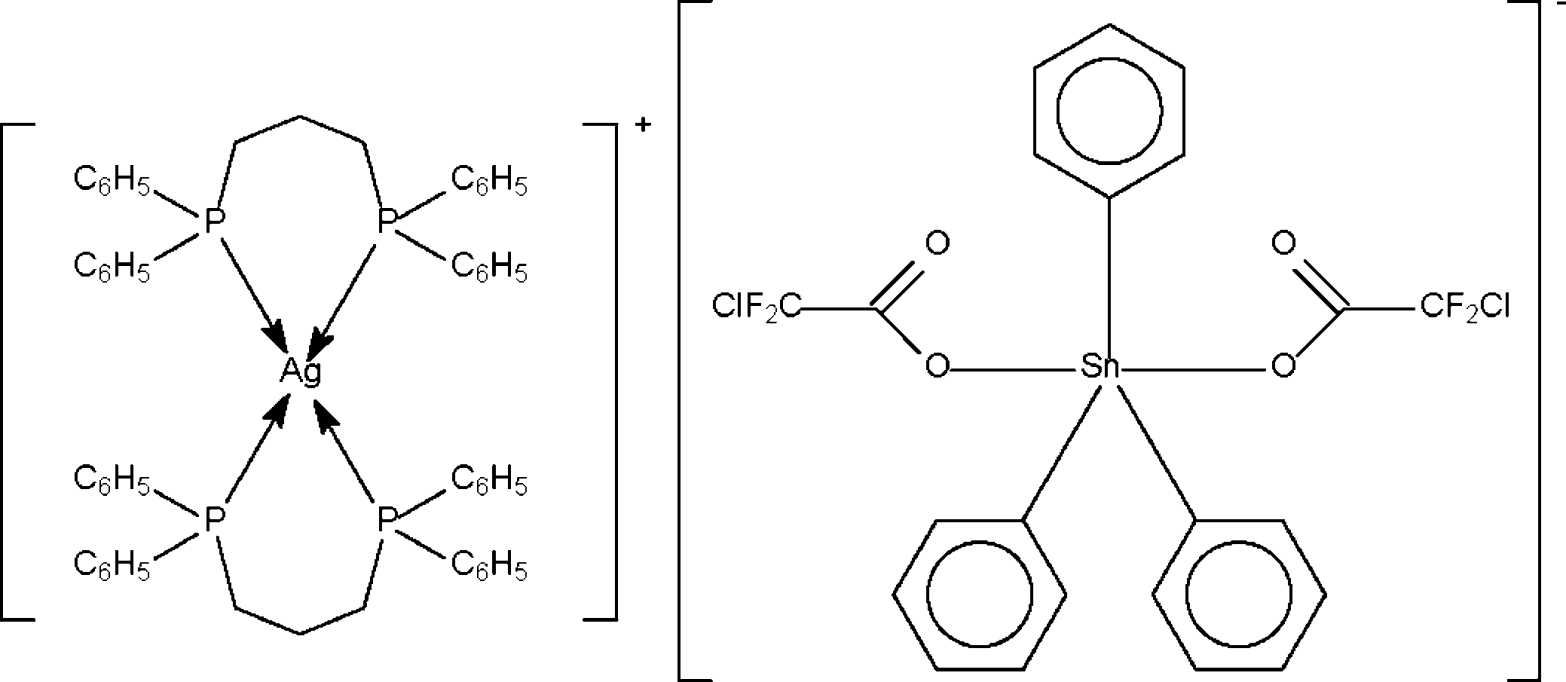


## Experimental

### 

#### Crystal data

[Ag(C_27_H_26_P_2_)_2_][Sn(C_6_H_5_)_3_(C_2_ClF_2_O_2_)]
*M* *_r_* = 1541.64
Triclinic, 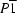

*a* = 10.5554 (2) Å
*b* = 17.6600 (4) Å
*c* = 19.1383 (4) Å
α = 91.581 (1)°
β = 94.931 (1)°
γ = 104.535 (1)°
*V* = 3436.1 (1) Å^3^
*Z* = 2
Mo *K*α radiation
μ = 0.88 mm^−1^
*T* = 100 (2) K
0.18 × 0.12 × 0.05 mm

#### Data collection

Bruker SMART APEXII diffractometer
Absorption correction: multi-scan (*SADABS*; Sheldrick, 1996 ▶) *T*_min_ = 0.858, *T*_max_ = 0.957
35712 measured reflections
15398 independent reflections
10993 reflections with *I* > 2σ(*I*)
*R*_int_ = 0.065

#### Refinement

*R*[*F* ^2^ > 2σ(*F* ^2^)] = 0.049
*wR*(*F* ^2^) = 0.112
*S* = 1.05
15398 reflections
829 parameters
48 restraints
H-atom parameters constrained
Δρ_max_ = 0.86 e Å^−3^
Δρ_min_ = −0.76 e Å^−3^

### 

Data collection: *APEX2* (Bruker, 2007 ▶); cell refinement: *SAINT* (Bruker, 2007 ▶); data reduction: *SAINT*; program(s) used to solve structure: *SHELXS97* (Sheldrick, 2008 ▶); program(s) used to refine structure: *SHELXL97* (Sheldrick, 2008 ▶); molecular graphics: *X-SEED* (Barbour, 2001 ▶); software used to prepare material for publication: *publCIF* (Westrip, 2008 ▶).

## Supplementary Material

Crystal structure: contains datablocks global, I. DOI: 10.1107/S1600536808010763/cv2399sup1.cif

Structure factors: contains datablocks I. DOI: 10.1107/S1600536808010763/cv2399Isup2.hkl

Additional supplementary materials:  crystallographic information; 3D view; checkCIF report

## Figures and Tables

**Fig. 1.**
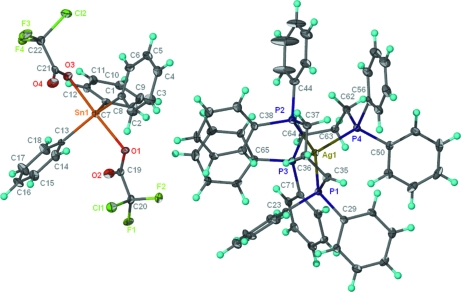
The 70% probability displacement ellipsoids plot (Barbour, 2001) of (I). Hydrogen atoms are drawn as spheres of arbitrary radii.

## supplementary crystallographic information

### Comment

The structural chemistry of di(carboxylato)triorganostannates has been reviewed by Tiekink (1991, 1994). This study continues our studies on bis(chlorodifluoroacetato)triorganostannates (Ng & Hook, 1999; Teo *et al.*, 2004, 2007, 2008). One of these studies used the bis[1,3-bis(diphenylphosphine)ethane]silver cation as counterion. Herewith we present the crystal structure of the title compound, (I), where [Ag(Ph_2_(CH_2_)_3_Ph_2_)_2_] cation is the counterion for [SnPh_3_(ClF_2_CCO_2_)_2_] anion (Scheme I).

In (I) (Fig. 1), the silver(I) and tin(IV) atoms show tetrahedral and *trans*-trigonal bipyramidal coordinations, respectively.

### Experimental

Triphenyltin hydroxide (0.18 g, 0.5 mmol) and chorodifluoroacetic acid (0.05 ml, 0.5 mmol) were dissolved in dichloromethane/methanol (25 ml). The mixture was heated until the hydroxide dissolved completely. Another solution containing 1,3-bis(diphenylphosphino)propane (0.41 g, 1.0 mmol) and silver trifluoroacetate (0.11 g, 0.5 mmol) was prepared; this was also heated until the reagents dissolved completely. The two solutions were mixed; crystals were obtained by allowing the solvent to evaporate in about 70% yield.

### Refinement

Carbon-bound H-atoms were placed in calculated positions (C—H 0.95 to 0.99 Å) and were included in the refinement in the riding model approximation, with *U*(H) set to 1.2*U*_eq_(C).

One of the phenyl rings shows large displacement ellipsoids. This ring, C44-C49, was restrained to be nearly planar; the bonded C-atoms were restrained to 1.39 (1) Å and 1,4-related ones to 2.78 (1) Å. The anisotropic displacement parameters of atoms C44-C49 were restrained to be nearly isotropic.

### Crystal data

**Table:**

[Ag(C_27_H_26_P_2_)_2_][Sn(C_6_H_5_)_3_(C_2_ClF_2_O_2_)]	*Z* = 2
*M**_r_* = 1541.64	*F*_000_ = 1564
Triclinic, *P*1	*D*_x_ = 1.490 Mg m^−^^3^
Hall symbol: -P 1	Mo *K*α radiation λ = 0.71073 Å
*a* = 10.5554 (2) Å	Cell parameters from 4777 reflections
*b* = 17.6600 (4) Å	θ = 2.2–24º
*c* = 19.1383 (4) Å	µ = 0.88 mm^−^^1^
α = 91.581 (1)º	*T* = 100 (2) K
β = 94.931 (1)º	Block, colourless
γ = 104.535 (1)º	0.18 × 0.12 × 0.05 mm
*V* = 3436.1 (1) Å^3^	

### Data collection

**Table:**

Bruker SMART APEX diffractometer	15398 independent reflections
Radiation source: fine-focus sealed tube	10993 reflections with *I* > 2σ(*I*)
Monochromator: graphite	*R*_int_ = 0.065
*T* = 100(2) K	θ_max_ = 27.5º
ω scans	θ_min_ = 1.1º
Absorption correction: multi-scan(SADABS; Sheldrick, 1996)	*h* = −13→13
*T*_min_ = 0.858, *T*_max_ = 0.957	*k* = −22→18
35712 measured reflections	*l* = −24→24

### Refinement

**Table:**

Refinement on *F*^2^	Secondary atom site location: difference Fourier map
Least-squares matrix: full	Hydrogen site location: inferred from neighbouring sites
*R*[*F*^2^ > 2σ(*F*^2^)] = 0.049	H-atom parameters constrained
*wR*(*F*^2^) = 0.112	*w* = 1/[σ^2^(*F*_o_^2^) + 1.3097*P*] where *P* = (*F*_o_^2^ + 2*F*_c_^2^)/3
*S* = 1.05	(Δ/σ)_max_ = 0.001
15398 reflections	Δρ_max_ = 0.86 e Å^−^^3^
829 parameters	Δρ_min_ = −0.76 e Å^−^^3^
48 restraints	Extinction correction: none
Primary atom site location: structure-invariant direct methods	

### Fractional atomic coordinates and isotropic or equivalent isotropic displacement parameters (Å^2^)

**Table:**

	*x*	*y*	*z*	*U*_iso_*/*U*_eq_	
Sn1	0.02753 (3)	0.730942 (18)	0.273935 (14)	0.01420 (8)	
Ag1	0.41787 (3)	0.227571 (19)	0.254989 (15)	0.01370 (8)	
Cl1	−0.23532 (11)	0.56238 (7)	0.09181 (6)	0.0289 (3)	
Cl2	0.08334 (11)	0.88104 (8)	0.50499 (6)	0.0294 (3)	
P1	0.21513 (10)	0.14129 (7)	0.19532 (5)	0.0140 (2)	
P2	0.30050 (10)	0.27516 (7)	0.34838 (5)	0.0164 (2)	
P3	0.56274 (10)	0.32427 (7)	0.18763 (6)	0.0154 (2)	
P4	0.59289 (10)	0.17736 (7)	0.31680 (6)	0.0153 (2)	
F1	−0.0366 (3)	0.56467 (16)	0.02666 (12)	0.0289 (6)	
F2	−0.0553 (3)	0.49132 (16)	0.11555 (13)	0.0301 (6)	
F3	−0.1207 (3)	0.92144 (19)	0.46015 (14)	0.0399 (8)	
F4	0.0540 (3)	0.97475 (16)	0.41018 (14)	0.0335 (7)	
O1	−0.0013 (3)	0.62288 (17)	0.20477 (14)	0.0173 (6)	
O2	0.0963 (3)	0.68053 (19)	0.11296 (15)	0.0253 (7)	
O3	0.0474 (3)	0.83204 (18)	0.34826 (14)	0.0202 (7)	
O4	−0.1681 (3)	0.8079 (2)	0.36239 (16)	0.0278 (8)	
C1	−0.0392 (4)	0.6519 (3)	0.3528 (2)	0.0168 (9)	
C2	−0.1432 (4)	0.5862 (3)	0.3396 (2)	0.0245 (10)	
H2	−0.1891	0.5759	0.2940	0.029*	
C3	−0.1815 (4)	0.5347 (3)	0.3923 (2)	0.0291 (11)	
H3	−0.2541	0.4902	0.3829	0.035*	
C4	−0.1132 (5)	0.5486 (3)	0.4585 (2)	0.0265 (11)	
H4	−0.1382	0.5136	0.4947	0.032*	
C5	−0.0085 (5)	0.6138 (3)	0.4714 (2)	0.0277 (11)	
H5	0.0394	0.6232	0.5164	0.033*	
C6	0.0270 (4)	0.6650 (3)	0.4197 (2)	0.0237 (10)	
H6	0.0982	0.7101	0.4297	0.028*	
C7	0.2352 (4)	0.7673 (3)	0.2721 (2)	0.0147 (9)	
C8	0.3074 (4)	0.7132 (3)	0.2625 (2)	0.0228 (10)	
H8	0.2638	0.6592	0.2548	0.027*	
C9	0.4436 (4)	0.7373 (3)	0.2641 (2)	0.0243 (10)	
H9	0.4927	0.6994	0.2599	0.029*	
C10	0.5081 (4)	0.8157 (3)	0.2717 (2)	0.0223 (10)	
H10	0.6009	0.8319	0.2714	0.027*	
C11	0.4365 (4)	0.8706 (3)	0.2799 (2)	0.0211 (10)	
H11	0.4795	0.9249	0.2843	0.025*	
C12	0.3011 (4)	0.8455 (3)	0.2815 (2)	0.0185 (9)	
H12	0.2526	0.8831	0.2893	0.022*	
C13	−0.0893 (4)	0.7739 (3)	0.1972 (2)	0.0152 (9)	
C14	−0.2241 (4)	0.7448 (3)	0.1842 (2)	0.0241 (10)	
H14	−0.2683	0.7057	0.2130	0.029*	
C15	−0.2964 (4)	0.7710 (3)	0.1308 (2)	0.0263 (11)	
H15	−0.3890	0.7504	0.1236	0.032*	
C16	−0.2335 (4)	0.8270 (3)	0.0883 (2)	0.0286 (12)	
H16	−0.2824	0.8442	0.0507	0.034*	
C17	−0.0999 (5)	0.8582 (3)	0.1001 (2)	0.0344 (13)	
H17	−0.0568	0.8977	0.0712	0.041*	
C18	−0.0276 (4)	0.8320 (3)	0.1543 (2)	0.0277 (11)	
H18	0.0646	0.8538	0.1622	0.033*	
C19	0.0206 (4)	0.6297 (3)	0.1401 (2)	0.0171 (9)	
C20	−0.0680 (4)	0.5614 (3)	0.0928 (2)	0.0212 (10)	
C21	−0.0532 (4)	0.8413 (3)	0.3756 (2)	0.0185 (9)	
C22	−0.0145 (4)	0.9070 (3)	0.4342 (2)	0.0228 (10)	
C23	0.1159 (4)	0.1904 (3)	0.1391 (2)	0.0155 (9)	
C24	0.1817 (4)	0.2448 (3)	0.0947 (2)	0.0197 (10)	
H24	0.2749	0.2567	0.0967	0.024*	
C25	0.1131 (4)	0.2815 (3)	0.0481 (2)	0.0256 (11)	
H25	0.1588	0.3180	0.0175	0.031*	
C26	−0.0226 (4)	0.2651 (3)	0.0458 (2)	0.0264 (11)	
H26	−0.0700	0.2904	0.0136	0.032*	
C27	−0.0893 (4)	0.2119 (3)	0.0902 (2)	0.0229 (10)	
H27	−0.1823	0.2017	0.0892	0.028*	
C28	−0.0209 (4)	0.1735 (3)	0.1364 (2)	0.0192 (9)	
H28	−0.0670	0.1360	0.1660	0.023*	
C29	0.2263 (4)	0.0595 (3)	0.1382 (2)	0.0158 (9)	
C30	0.1691 (4)	0.0471 (3)	0.0691 (2)	0.0195 (10)	
H30	0.1166	0.0800	0.0512	0.023*	
C31	0.1881 (4)	−0.0129 (3)	0.0260 (2)	0.0251 (11)	
H31	0.1506	−0.0197	−0.0214	0.030*	
C32	0.2605 (4)	−0.0625 (3)	0.0513 (2)	0.0246 (10)	
H32	0.2705	−0.1045	0.0220	0.030*	
C33	0.3193 (4)	−0.0510 (3)	0.1201 (2)	0.0249 (11)	
H33	0.3708	−0.0846	0.1376	0.030*	
C34	0.3023 (4)	0.0096 (3)	0.1626 (2)	0.0223 (10)	
H34	0.3431	0.0174	0.2094	0.027*	
C35	0.1064 (4)	0.0964 (3)	0.2607 (2)	0.0182 (9)	
H35	0.0774	0.0413	0.2641	0.022*	
C36	0.0654 (4)	0.1549 (3)	0.3099 (2)	0.0204 (10)	
H36A	0.0431	0.1963	0.2810	0.025*	
H36B	−0.0159	0.1269	0.3298	0.025*	
C37	0.1649 (4)	0.1948 (3)	0.3706 (2)	0.0193 (10)	
H37A	0.2029	0.1546	0.3931	0.023*	
H37B	0.1172	0.2154	0.4060	0.023*	
C38	0.2178 (4)	0.3504 (3)	0.3206 (2)	0.0174 (9)	
C39	0.2053 (4)	0.3658 (3)	0.2501 (2)	0.0208 (10)	
H39	0.2414	0.3380	0.2171	0.025*	
C40	0.1405 (4)	0.4214 (3)	0.2269 (2)	0.0220 (10)	
H40	0.1303	0.4303	0.1782	0.026*	
C41	0.0911 (4)	0.4635 (3)	0.2747 (2)	0.0260 (11)	
H41	0.0482	0.5022	0.2593	0.031*	
C42	0.1047 (5)	0.4489 (3)	0.3456 (2)	0.0372 (14)	
H42	0.0715	0.4781	0.3788	0.045*	
C43	0.1659 (5)	0.3927 (3)	0.3679 (2)	0.0324 (13)	
H43	0.1728	0.3825	0.4164	0.039*	
C44	0.3843 (4)	0.3140 (3)	0.4337 (2)	0.0210 (10)	
C45	0.4909 (5)	0.3775 (3)	0.4357 (2)	0.0566 (18)	
H45A	0.4607	0.4195	0.4114	0.068*	
H45B	0.5550	0.3625	0.4069	0.068*	
C46	0.5602 (5)	0.4111 (3)	0.4991 (3)	0.085 (3)	
H46	0.6336	0.4552	0.4994	0.102*	
C47	0.5213 (5)	0.3798 (3)	0.5610 (2)	0.0516 (16)	
H47	0.5683	0.4022	0.6044	0.062*	
C48	0.4156 (4)	0.3168 (3)	0.5608 (2)	0.0359 (13)	
H48	0.3890	0.2955	0.6038	0.043*	
C49	0.3468 (4)	0.2837 (3)	0.4974 (2)	0.0298 (11)	
H49	0.2733	0.2398	0.4976	0.036*	
C50	0.6527 (4)	0.0962 (3)	0.2826 (2)	0.0179 (9)	
C51	0.6416 (4)	0.0806 (3)	0.2098 (2)	0.0214 (10)	
H51	0.5998	0.1103	0.1792	0.026*	
C52	0.6915 (4)	0.0218 (3)	0.1824 (3)	0.0275 (11)	
H52	0.6850	0.0119	0.1331	0.033*	
C53	0.7504 (4)	−0.0222 (3)	0.2263 (3)	0.0293 (12)	
H53	0.7842	−0.0624	0.2071	0.035*	
C54	0.7605 (4)	−0.0080 (3)	0.2983 (3)	0.0295 (12)	
H54	0.8009	−0.0388	0.3285	0.035*	
C55	0.7116 (4)	0.0513 (3)	0.3266 (2)	0.0220 (10)	
H55	0.7188	0.0609	0.3761	0.026*	
C56	0.5544 (4)	0.1494 (3)	0.4043 (2)	0.0180 (9)	
C57	0.4425 (4)	0.0884 (3)	0.4092 (2)	0.0232 (10)	
H57	0.3946	0.0617	0.3676	0.028*	
C58	0.4006 (4)	0.0663 (3)	0.4738 (2)	0.0278 (11)	
H58	0.3255	0.0239	0.4763	0.033*	
C59	0.4674 (5)	0.1057 (3)	0.5348 (2)	0.0315 (12)	
H59	0.4372	0.0911	0.5791	0.038*	
C60	0.5778 (5)	0.1660 (3)	0.5311 (2)	0.0327 (12)	
H60	0.6246	0.1928	0.5730	0.039*	
C61	0.6211 (4)	0.1882 (3)	0.4658 (2)	0.0245 (10)	
H61	0.6970	0.2301	0.4637	0.029*	
C62	0.7435 (4)	0.2573 (3)	0.3283 (2)	0.0179 (9)	
H62A	0.7277	0.3002	0.3579	0.022*	
H62B	0.8141	0.2381	0.3536	0.022*	
C63	0.7910 (4)	0.2905 (3)	0.2587 (2)	0.0165 (9)	
H63A	0.8878	0.3107	0.2652	0.020*	
H63B	0.7703	0.2470	0.2224	0.020*	
C64	0.7316 (4)	0.3561 (3)	0.2311 (2)	0.0183 (9)	
H64A	0.7893	0.3852	0.1974	0.022*	
H64B	0.7321	0.3931	0.2710	0.022*	
C65	0.5173 (4)	0.4162 (3)	0.1735 (2)	0.0160 (9)	
C66	0.4562 (4)	0.4311 (3)	0.1095 (2)	0.0223 (10)	
H66	0.4427	0.3941	0.0709	0.027*	
C67	0.4145 (4)	0.5001 (3)	0.1019 (2)	0.0245 (11)	
H67	0.3748	0.5105	0.0580	0.029*	
C68	0.4318 (5)	0.5522 (3)	0.1582 (3)	0.0305 (12)	
H68	0.4023	0.5985	0.1533	0.037*	
C69	0.4916 (5)	0.5385 (3)	0.2223 (2)	0.0310 (12)	
H69	0.5044	0.5755	0.2609	0.037*	
C70	0.5325 (4)	0.4702 (3)	0.2293 (2)	0.0227 (10)	
H70	0.5720	0.4603	0.2734	0.027*	
C71	0.5872 (4)	0.2907 (3)	0.0999 (2)	0.0154 (9)	
C72	0.6675 (4)	0.3406 (3)	0.0568 (2)	0.0187 (9)	
H72	0.7113	0.3925	0.0735	0.022*	
C73	0.6833 (4)	0.3150 (3)	−0.0097 (2)	0.0214 (10)	
H73	0.7363	0.3496	−0.0390	0.026*	
C74	0.6222 (4)	0.2389 (3)	−0.0339 (2)	0.0245 (11)	
H74	0.6349	0.2212	−0.0794	0.029*	
C75	0.5432 (4)	0.1889 (3)	0.0076 (2)	0.0225 (10)	
H75	0.5011	0.1367	−0.0092	0.027*	
C76	0.5250 (4)	0.2150 (3)	0.0746 (2)	0.0180 (9)	
H76	0.4696	0.1807	0.1031	0.022*	

### Atomic displacement parameters (Å^2^)

**Table:**

	*U*^11^	*U*^22^	*U*^33^	*U*^12^	*U*^13^	*U*^23^
Sn1	0.01322 (14)	0.01595 (17)	0.01312 (15)	0.00299 (12)	0.00177 (11)	0.00034 (12)
Ag1	0.01216 (15)	0.01514 (18)	0.01399 (16)	0.00362 (13)	0.00165 (12)	0.00158 (13)
Cl1	0.0229 (6)	0.0264 (7)	0.0336 (7)	0.0011 (5)	−0.0020 (5)	0.0000 (5)
Cl2	0.0327 (6)	0.0326 (7)	0.0199 (6)	0.0051 (5)	−0.0038 (5)	−0.0009 (5)
P1	0.0130 (5)	0.0145 (6)	0.0143 (5)	0.0029 (4)	0.0007 (4)	0.0020 (5)
P2	0.0162 (5)	0.0194 (6)	0.0145 (5)	0.0057 (5)	0.0034 (4)	0.0006 (5)
P3	0.0160 (5)	0.0140 (6)	0.0155 (6)	0.0025 (5)	0.0017 (4)	0.0011 (5)
P4	0.0131 (5)	0.0161 (6)	0.0164 (6)	0.0036 (5)	0.0001 (4)	0.0023 (5)
F1	0.0449 (16)	0.0296 (17)	0.0143 (13)	0.0131 (14)	0.0064 (12)	−0.0045 (12)
F2	0.0426 (16)	0.0179 (16)	0.0309 (15)	0.0121 (13)	−0.0032 (12)	0.0003 (12)
F3	0.0325 (16)	0.055 (2)	0.0360 (17)	0.0216 (15)	0.0027 (13)	−0.0212 (15)
F4	0.0470 (17)	0.0209 (17)	0.0308 (16)	0.0071 (14)	−0.0009 (13)	−0.0018 (13)
O1	0.0225 (15)	0.0171 (17)	0.0119 (14)	0.0036 (13)	0.0036 (12)	−0.0004 (13)
O2	0.0261 (17)	0.026 (2)	0.0215 (17)	−0.0005 (15)	0.0089 (13)	0.0020 (15)
O3	0.0196 (15)	0.0210 (18)	0.0205 (16)	0.0057 (13)	0.0043 (12)	−0.0019 (14)
O4	0.0175 (16)	0.034 (2)	0.0298 (18)	0.0040 (15)	0.0013 (13)	−0.0066 (16)
C1	0.019 (2)	0.020 (3)	0.013 (2)	0.0068 (19)	0.0038 (16)	0.0006 (19)
C2	0.025 (2)	0.029 (3)	0.017 (2)	0.003 (2)	0.0011 (18)	0.002 (2)
C3	0.025 (2)	0.026 (3)	0.031 (3)	−0.005 (2)	0.005 (2)	0.005 (2)
C4	0.037 (3)	0.027 (3)	0.020 (2)	0.013 (2)	0.012 (2)	0.010 (2)
C5	0.036 (3)	0.028 (3)	0.019 (2)	0.008 (2)	−0.002 (2)	0.006 (2)
C6	0.028 (2)	0.022 (3)	0.019 (2)	0.003 (2)	−0.0019 (19)	−0.003 (2)
C7	0.0094 (18)	0.018 (2)	0.014 (2)	−0.0011 (17)	−0.0001 (15)	0.0000 (18)
C8	0.018 (2)	0.018 (3)	0.031 (3)	0.0037 (19)	0.0032 (19)	−0.004 (2)
C9	0.016 (2)	0.019 (3)	0.038 (3)	0.007 (2)	0.0031 (19)	−0.003 (2)
C10	0.014 (2)	0.028 (3)	0.024 (2)	0.005 (2)	0.0010 (18)	0.000 (2)
C11	0.017 (2)	0.017 (3)	0.027 (2)	−0.0002 (19)	−0.0009 (18)	0.003 (2)
C12	0.017 (2)	0.017 (2)	0.023 (2)	0.0062 (19)	0.0043 (17)	0.004 (2)
C13	0.019 (2)	0.014 (2)	0.012 (2)	0.0052 (18)	−0.0009 (16)	−0.0007 (18)
C14	0.020 (2)	0.021 (3)	0.032 (3)	0.004 (2)	0.0048 (19)	0.009 (2)
C15	0.014 (2)	0.028 (3)	0.035 (3)	0.004 (2)	−0.0036 (19)	0.003 (2)
C16	0.026 (2)	0.040 (3)	0.022 (2)	0.014 (2)	−0.0053 (19)	0.004 (2)
C17	0.027 (3)	0.044 (4)	0.032 (3)	0.006 (2)	0.003 (2)	0.021 (3)
C18	0.020 (2)	0.030 (3)	0.030 (3)	0.000 (2)	0.0010 (19)	0.013 (2)
C19	0.020 (2)	0.018 (3)	0.015 (2)	0.0077 (19)	0.0034 (17)	0.0015 (19)
C20	0.027 (2)	0.014 (2)	0.023 (2)	0.006 (2)	0.0040 (19)	0.003 (2)
C21	0.025 (2)	0.020 (3)	0.014 (2)	0.010 (2)	0.0025 (17)	0.0058 (19)
C22	0.020 (2)	0.022 (3)	0.027 (3)	0.008 (2)	−0.0014 (19)	−0.002 (2)
C23	0.019 (2)	0.013 (2)	0.014 (2)	0.0039 (18)	−0.0011 (16)	−0.0006 (18)
C24	0.017 (2)	0.022 (3)	0.021 (2)	0.0057 (19)	0.0027 (17)	0.002 (2)
C25	0.028 (2)	0.026 (3)	0.027 (3)	0.011 (2)	0.005 (2)	0.015 (2)
C26	0.024 (2)	0.038 (3)	0.022 (2)	0.016 (2)	0.0017 (19)	0.004 (2)
C27	0.018 (2)	0.036 (3)	0.016 (2)	0.012 (2)	−0.0037 (17)	−0.002 (2)
C28	0.018 (2)	0.020 (3)	0.018 (2)	0.0033 (19)	0.0011 (17)	0.000 (2)
C29	0.017 (2)	0.016 (2)	0.013 (2)	0.0013 (18)	0.0039 (16)	0.0049 (18)
C30	0.018 (2)	0.020 (3)	0.022 (2)	0.0084 (19)	−0.0018 (17)	0.001 (2)
C31	0.027 (2)	0.031 (3)	0.018 (2)	0.010 (2)	−0.0003 (19)	0.001 (2)
C32	0.027 (2)	0.020 (3)	0.027 (3)	0.006 (2)	0.006 (2)	−0.007 (2)
C33	0.025 (2)	0.021 (3)	0.031 (3)	0.010 (2)	0.004 (2)	0.003 (2)
C34	0.021 (2)	0.027 (3)	0.019 (2)	0.010 (2)	−0.0058 (18)	0.000 (2)
C35	0.022 (2)	0.014 (2)	0.018 (2)	−0.0008 (18)	0.0095 (17)	0.0034 (19)
C36	0.017 (2)	0.026 (3)	0.018 (2)	0.0040 (19)	0.0037 (17)	0.002 (2)
C37	0.023 (2)	0.024 (3)	0.012 (2)	0.009 (2)	0.0033 (17)	0.0010 (19)
C38	0.020 (2)	0.018 (2)	0.016 (2)	0.0072 (19)	0.0037 (17)	0.0048 (19)
C39	0.023 (2)	0.024 (3)	0.017 (2)	0.008 (2)	0.0058 (18)	0.000 (2)
C40	0.028 (2)	0.024 (3)	0.013 (2)	0.006 (2)	−0.0013 (18)	0.001 (2)
C41	0.028 (2)	0.018 (3)	0.036 (3)	0.011 (2)	0.004 (2)	0.008 (2)
C42	0.061 (4)	0.038 (4)	0.024 (3)	0.033 (3)	0.004 (2)	−0.003 (2)
C43	0.052 (3)	0.037 (3)	0.018 (2)	0.026 (3)	0.010 (2)	0.007 (2)
C44	0.021 (2)	0.029 (3)	0.014 (2)	0.008 (2)	0.0034 (17)	−0.0028 (19)
C45	0.056 (4)	0.068 (5)	0.026 (3)	−0.022 (3)	0.007 (3)	−0.002 (3)
C46	0.073 (4)	0.108 (6)	0.034 (3)	−0.047 (4)	0.003 (3)	−0.011 (4)
C47	0.045 (3)	0.072 (5)	0.023 (3)	−0.007 (3)	−0.003 (2)	−0.010 (3)
C48	0.045 (3)	0.040 (3)	0.021 (3)	0.008 (3)	0.001 (2)	0.008 (2)
C49	0.037 (3)	0.032 (3)	0.019 (2)	0.008 (2)	−0.001 (2)	0.003 (2)
C50	0.0111 (19)	0.015 (2)	0.027 (2)	0.0020 (17)	0.0006 (17)	0.0003 (19)
C51	0.019 (2)	0.023 (3)	0.024 (2)	0.004 (2)	0.0077 (18)	0.006 (2)
C52	0.029 (3)	0.019 (3)	0.032 (3)	−0.001 (2)	0.014 (2)	−0.005 (2)
C53	0.019 (2)	0.015 (3)	0.053 (3)	0.000 (2)	0.011 (2)	−0.007 (2)
C54	0.012 (2)	0.016 (3)	0.059 (4)	0.0017 (19)	−0.004 (2)	0.005 (2)
C55	0.013 (2)	0.025 (3)	0.025 (2)	0.0022 (19)	−0.0049 (17)	0.001 (2)
C56	0.014 (2)	0.021 (3)	0.022 (2)	0.0112 (19)	0.0004 (17)	0.007 (2)
C57	0.021 (2)	0.024 (3)	0.024 (2)	0.006 (2)	−0.0025 (18)	0.004 (2)
C58	0.026 (2)	0.029 (3)	0.031 (3)	0.007 (2)	0.011 (2)	0.018 (2)
C59	0.040 (3)	0.040 (3)	0.018 (2)	0.013 (3)	0.010 (2)	0.011 (2)
C60	0.040 (3)	0.039 (3)	0.020 (3)	0.010 (3)	0.004 (2)	0.007 (2)
C61	0.025 (2)	0.026 (3)	0.020 (2)	0.003 (2)	0.0018 (19)	0.008 (2)
C62	0.019 (2)	0.016 (2)	0.018 (2)	0.0033 (18)	−0.0002 (17)	0.0016 (19)
C63	0.0113 (19)	0.017 (2)	0.020 (2)	0.0021 (17)	0.0008 (16)	−0.0005 (19)
C64	0.020 (2)	0.018 (2)	0.014 (2)	0.0021 (19)	0.0011 (17)	−0.0015 (19)
C65	0.016 (2)	0.016 (2)	0.015 (2)	0.0003 (18)	0.0032 (16)	0.0011 (18)
C66	0.022 (2)	0.022 (3)	0.020 (2)	0.002 (2)	0.0018 (18)	−0.008 (2)
C67	0.030 (2)	0.020 (3)	0.026 (3)	0.012 (2)	0.000 (2)	−0.001 (2)
C68	0.038 (3)	0.021 (3)	0.036 (3)	0.012 (2)	0.005 (2)	0.006 (2)
C69	0.046 (3)	0.026 (3)	0.022 (3)	0.012 (3)	0.002 (2)	−0.004 (2)
C70	0.031 (2)	0.016 (3)	0.021 (2)	0.006 (2)	0.0006 (19)	0.000 (2)
C71	0.0111 (19)	0.019 (2)	0.016 (2)	0.0042 (18)	−0.0014 (16)	0.0001 (19)
C72	0.018 (2)	0.017 (2)	0.021 (2)	0.0038 (19)	0.0001 (17)	0.005 (2)
C73	0.021 (2)	0.028 (3)	0.021 (2)	0.012 (2)	0.0069 (18)	0.008 (2)
C74	0.025 (2)	0.039 (3)	0.017 (2)	0.022 (2)	0.0037 (18)	0.001 (2)
C75	0.022 (2)	0.023 (3)	0.024 (2)	0.013 (2)	−0.0079 (18)	−0.010 (2)
C76	0.0110 (19)	0.021 (3)	0.022 (2)	0.0046 (18)	0.0010 (17)	0.002 (2)

### Geometric parameters (Å, °)

**Table:**

Sn1—C1	2.128 (4)	C33—C34	1.384 (6)
Sn1—C7	2.128 (4)	C33—H33	0.9500
Sn1—C13	2.115 (4)	C34—H34	0.9500
Sn1—O1	2.233 (3)	C35—C36	1.543 (6)
Sn1—O3	2.208 (3)	C35—H35	0.9500
Ag1—P1	2.460 (1)	C36—C37	1.524 (6)
Ag1—P2	2.501 (1)	C36—H36A	0.9900
Ag1—P3	2.463 (1)	C36—H36B	0.9900
Ag1—P4	2.470 (1)	C37—H37A	0.9900
Cl1—C20	1.769 (4)	C37—H37B	0.9900
Cl2—C22	1.773 (4)	C38—C39	1.385 (5)
P1—C29	1.821 (4)	C38—C43	1.390 (6)
P1—C23	1.822 (4)	C39—C40	1.392 (6)
P1—C35	1.833 (4)	C39—H39	0.9500
P2—C44	1.818 (4)	C40—C41	1.379 (6)
P2—C38	1.831 (4)	C40—H40	0.9500
P2—C37	1.834 (5)	C41—C42	1.390 (6)
P3—C65	1.825 (4)	C41—H41	0.9500
P3—C71	1.827 (4)	C42—C43	1.373 (6)
P3—C64	1.845 (4)	C42—H42	0.9500
P4—C56	1.810 (4)	C43—H43	0.9500
P4—C50	1.831 (4)	C44—C45	1.373 (6)
P4—C62	1.835 (4)	C44—C49	1.394 (6)
F1—C20	1.334 (5)	C45—C46	1.396 (6)
F2—C20	1.358 (5)	C45—H45A	0.9900
F3—C22	1.342 (4)	C45—H45B	0.9900
F4—C22	1.348 (5)	C46—C47	1.373 (6)
O1—C19	1.282 (4)	C46—H46	0.9500
O2—C19	1.204 (5)	C47—C48	1.363 (6)
O3—C21	1.268 (4)	C47—H47	0.9500
O4—C21	1.209 (5)	C48—C49	1.391 (5)
C1—C2	1.382 (6)	C48—H48	0.9500
C1—C6	1.390 (6)	C49—H49	0.9500
C2—C3	1.395 (6)	C50—C55	1.386 (5)
C2—H2	0.9500	C50—C51	1.403 (6)
C3—C4	1.386 (6)	C51—C52	1.386 (6)
C3—H3	0.9500	C51—H51	0.9500
C4—C5	1.381 (7)	C52—C53	1.371 (6)
C4—H4	0.9500	C52—H52	0.9500
C5—C6	1.371 (6)	C53—C54	1.385 (7)
C5—H5	0.9500	C53—H53	0.9500
C6—H6	0.9500	C54—C55	1.396 (6)
C7—C12	1.380 (6)	C54—H54	0.9500
C7—C8	1.382 (5)	C55—H55	0.9500
C8—C9	1.391 (6)	C56—C61	1.386 (6)
C8—H8	0.9500	C56—C57	1.396 (6)
C9—C10	1.378 (6)	C57—C58	1.381 (6)
C9—H9	0.9500	C57—H57	0.9500
C10—C11	1.384 (6)	C58—C59	1.382 (7)
C10—H10	0.9500	C58—H58	0.9500
C11—C12	1.390 (5)	C59—C60	1.376 (7)
C11—H11	0.9500	C59—H59	0.9500
C12—H12	0.9500	C60—C61	1.399 (6)
C13—C14	1.384 (6)	C60—H60	0.9500
C13—C18	1.398 (6)	C61—H61	0.9500
C14—C15	1.382 (6)	C62—C63	1.542 (5)
C14—H14	0.9500	C62—H62A	0.9900
C15—C16	1.375 (7)	C62—H62B	0.9900
C15—H15	0.9500	C63—C64	1.535 (5)
C16—C17	1.376 (6)	C63—H63A	0.9900
C16—H16	0.9500	C63—H63B	0.9900
C17—C18	1.393 (6)	C64—H64A	0.9900
C17—H17	0.9500	C64—H64B	0.9900
C18—H18	0.9500	C65—C70	1.383 (6)
C19—C20	1.543 (6)	C65—C66	1.396 (6)
C21—C22	1.544 (6)	C66—C67	1.402 (6)
C23—C24	1.392 (6)	C66—H66	0.9500
C23—C28	1.394 (5)	C67—C68	1.368 (6)
C24—C25	1.378 (5)	C67—H67	0.9500
C24—H24	0.9500	C68—C69	1.386 (6)
C25—C26	1.385 (6)	C68—H68	0.9500
C25—H25	0.9500	C69—C70	1.385 (6)
C26—C27	1.385 (6)	C69—H69	0.9500
C26—H26	0.9500	C70—H70	0.9500
C27—C28	1.390 (5)	C71—C76	1.385 (6)
C27—H27	0.9500	C71—C72	1.400 (6)
C28—H28	0.9500	C72—C73	1.375 (6)
C29—C30	1.394 (6)	C72—H72	0.9500
C29—C34	1.396 (5)	C73—C74	1.384 (7)
C30—C31	1.389 (6)	C73—H73	0.9500
C30—H30	0.9500	C74—C75	1.375 (7)
C31—C32	1.371 (6)	C74—H74	0.9500
C31—H31	0.9500	C75—C76	1.396 (5)
C32—C33	1.393 (6)	C75—H75	0.9500
C32—H32	0.9500	C76—H76	0.9500
			
C1—Sn1—C7	115.2 (2)	C33—C34—C29	121.3 (4)
C1—Sn1—C13	127.2 (2)	C33—C34—H34	119.4
C1—Sn1—O1	84.9 (1)	C29—C34—H34	119.4
C1—Sn1—O3	90.8 (1)	C36—C35—P1	114.9 (3)
C7—Sn1—C13	117.6 (2)	C36—C35—H35	122.5
C7—Sn1—O1	95.8 (1)	P1—C35—H35	122.5
C7—Sn1—O3	86.9 (1)	C37—C36—C35	117.3 (3)
C13—Sn1—O3	93.2 (1)	C37—C36—H36A	108.0
C13—Sn1—O1	88.7 (1)	C35—C36—H36A	108.0
O1—Sn1—O3	175.6 (1)	C37—C36—H36B	108.0
P1—Ag1—P2	93.58 (4)	C35—C36—H36B	108.0
P1—Ag1—P3	119.54 (4)	H36A—C36—H36B	107.2
P1—Ag1—P4	122.91 (4)	C36—C37—P2	116.3 (3)
P2—Ag1—P3	118.95 (4)	C36—C37—H37A	108.2
P2—Ag1—P4	106.23 (4)	P2—C37—H37A	108.2
P3—Ag1—P4	96.64 (4)	C36—C37—H37B	108.2
C29—P1—C23	102.35 (18)	P2—C37—H37B	108.2
C29—P1—C35	104.7 (2)	H37A—C37—H37B	107.4
C23—P1—C35	104.52 (18)	C39—C38—C43	118.5 (4)
C29—P1—Ag1	119.20 (13)	C39—C38—P2	119.3 (3)
C23—P1—Ag1	115.03 (15)	C43—C38—P2	122.2 (3)
C35—P1—Ag1	109.63 (14)	C38—C39—C40	120.9 (4)
C44—P2—C38	103.21 (19)	C38—C39—H39	119.6
C44—P2—C37	103.13 (19)	C40—C39—H39	119.6
C38—P2—C37	103.20 (19)	C41—C40—C39	119.9 (4)
C44—P2—Ag1	121.75 (12)	C41—C40—H40	120.1
C38—P2—Ag1	114.10 (13)	C39—C40—H40	120.1
C37—P2—Ag1	109.46 (14)	C40—C41—C42	119.5 (4)
C65—P3—C71	103.91 (18)	C40—C41—H41	120.3
C65—P3—C64	103.5 (2)	C42—C41—H41	120.3
C71—P3—C64	103.10 (18)	C43—C42—C41	120.4 (4)
C65—P3—Ag1	116.75 (13)	C43—C42—H42	119.8
C71—P3—Ag1	115.99 (15)	C41—C42—H42	119.8
C64—P3—Ag1	112.00 (14)	C42—C43—C38	120.9 (4)
C56—P4—C50	103.42 (19)	C42—C43—H43	119.6
C56—P4—C62	106.1 (2)	C38—C43—H43	119.6
C50—P4—C62	102.09 (19)	C45—C44—C49	117.7 (4)
C56—P4—Ag1	111.49 (12)	C45—C44—P2	118.1 (3)
C50—P4—Ag1	124.14 (14)	C49—C44—P2	124.3 (3)
C62—P4—Ag1	108.08 (13)	C44—C45—C46	121.6 (4)
C19—O1—Sn1	119.0 (3)	C44—C45—H45A	106.9
C21—O3—Sn1	119.3 (3)	C46—C45—H45A	106.9
C2—C1—C6	118.3 (4)	C44—C45—H45B	106.9
C2—C1—Sn1	122.7 (3)	C46—C45—H45B	106.9
C6—C1—Sn1	118.9 (3)	H45A—C45—H45B	106.7
C1—C2—C3	120.9 (4)	C47—C46—C45	119.3 (5)
C1—C2—H2	119.6	C47—C46—H46	120.3
C3—C2—H2	119.6	C45—C46—H46	120.3
C4—C3—C2	119.8 (5)	C48—C47—C46	120.4 (5)
C4—C3—H3	120.1	C48—C47—H47	119.8
C2—C3—H3	120.1	C46—C47—H47	119.8
C5—C4—C3	119.3 (4)	C47—C48—C49	119.9 (4)
C5—C4—H4	120.4	C47—C48—H48	120.0
C3—C4—H4	120.4	C49—C48—H48	120.0
C6—C5—C4	120.6 (4)	C48—C49—C44	121.0 (4)
C6—C5—H5	119.7	C48—C49—H49	119.5
C4—C5—H5	119.7	C44—C49—H49	119.5
C5—C6—C1	121.1 (5)	C55—C50—C51	119.2 (4)
C5—C6—H6	119.5	C55—C50—P4	121.8 (3)
C1—C6—H6	119.5	C51—C50—P4	119.0 (3)
C12—C7—C8	118.5 (4)	C52—C51—C50	120.1 (4)
C12—C7—Sn1	120.7 (3)	C52—C51—H51	120.0
C8—C7—Sn1	120.8 (3)	C50—C51—H51	120.0
C7—C8—C9	120.3 (4)	C53—C52—C51	120.4 (4)
C7—C8—H8	119.8	C53—C52—H52	119.8
C9—C8—H8	119.8	C51—C52—H52	119.8
C10—C9—C8	120.7 (4)	C52—C53—C54	120.1 (4)
C10—C9—H9	119.7	C52—C53—H53	119.9
C8—C9—H9	119.7	C54—C53—H53	119.9
C9—C10—C11	119.4 (4)	C53—C54—C55	120.2 (4)
C9—C10—H10	120.3	C53—C54—H54	119.9
C11—C10—H10	120.3	C55—C54—H54	119.9
C10—C11—C12	119.3 (4)	C50—C55—C54	120.0 (4)
C10—C11—H11	120.3	C50—C55—H55	120.0
C12—C11—H11	120.3	C54—C55—H55	120.0
C7—C12—C11	121.6 (4)	C61—C56—C57	118.3 (4)
C7—C12—H12	119.2	C61—C56—P4	124.7 (4)
C11—C12—H12	119.2	C57—C56—P4	116.7 (3)
C14—C13—C18	117.4 (4)	C58—C57—C56	120.9 (4)
C14—C13—Sn1	123.6 (3)	C58—C57—H57	119.6
C18—C13—Sn1	118.9 (3)	C56—C57—H57	119.6
C15—C14—C13	122.1 (4)	C57—C58—C59	120.4 (5)
C15—C14—H14	118.9	C57—C58—H58	119.8
C13—C14—H14	118.9	C59—C58—H58	119.8
C16—C15—C14	119.6 (4)	C60—C59—C58	119.6 (4)
C16—C15—H15	120.2	C60—C59—H59	120.2
C14—C15—H15	120.2	C58—C59—H59	120.2
C15—C16—C17	120.1 (4)	C59—C60—C61	120.1 (5)
C15—C16—H16	120.0	C59—C60—H60	119.9
C17—C16—H16	120.0	C61—C60—H60	119.9
C16—C17—C18	120.1 (4)	C56—C61—C60	120.6 (5)
C16—C17—H17	120.0	C56—C61—H61	119.7
C18—C17—H17	120.0	C60—C61—H61	119.7
C17—C18—C13	120.7 (4)	C63—C62—P4	113.8 (3)
C17—C18—H18	119.6	C63—C62—H62A	108.8
C13—C18—H18	119.6	P4—C62—H62A	108.8
O2—C19—O1	129.8 (4)	C63—C62—H62B	108.8
O2—C19—C20	118.3 (4)	P4—C62—H62B	108.8
O1—C19—C20	111.9 (4)	H62A—C62—H62B	107.7
F1—C20—F2	106.7 (3)	C64—C63—C62	115.4 (3)
F1—C20—C19	111.8 (4)	C64—C63—H63A	108.4
F2—C20—C19	111.0 (3)	C62—C63—H63A	108.4
F1—C20—Cl1	108.0 (3)	C64—C63—H63B	108.4
F2—C20—Cl1	108.2 (3)	C62—C63—H63B	108.4
C19—C20—Cl1	111.0 (3)	H63A—C63—H63B	107.5
O4—C21—O3	130.4 (4)	C63—C64—P3	115.7 (3)
O4—C21—C22	118.8 (4)	C63—C64—H64A	108.4
O3—C21—C22	110.9 (4)	P3—C64—H64A	108.4
F3—C22—F4	107.1 (4)	C63—C64—H64B	108.4
F3—C22—C21	111.6 (4)	P3—C64—H64B	108.4
F4—C22—C21	111.4 (3)	H64A—C64—H64B	107.4
F3—C22—Cl2	107.4 (3)	C70—C65—C66	118.3 (4)
F4—C22—Cl2	108.2 (3)	C70—C65—P3	119.4 (3)
C21—C22—Cl2	110.9 (3)	C66—C65—P3	122.1 (3)
C24—C23—C28	119.4 (4)	C65—C66—C67	120.6 (4)
C24—C23—P1	117.1 (3)	C65—C66—H66	119.7
C28—C23—P1	123.4 (3)	C67—C66—H66	119.7
C25—C24—C23	120.7 (4)	C68—C67—C66	119.3 (4)
C25—C24—H24	119.6	C68—C67—H67	120.3
C23—C24—H24	119.6	C66—C67—H67	120.3
C24—C25—C26	119.8 (4)	C67—C68—C69	121.1 (4)
C24—C25—H25	120.1	C67—C68—H68	119.5
C26—C25—H25	120.1	C69—C68—H68	119.5
C27—C26—C25	120.1 (4)	C70—C69—C68	119.1 (5)
C27—C26—H26	119.9	C70—C69—H69	120.5
C25—C26—H26	119.9	C68—C69—H69	120.5
C26—C27—C28	120.3 (4)	C65—C70—C69	121.6 (4)
C26—C27—H27	119.9	C65—C70—H70	119.2
C28—C27—H27	119.9	C69—C70—H70	119.2
C27—C28—C23	119.6 (4)	C76—C71—C72	118.9 (4)
C27—C28—H28	120.2	C76—C71—P3	120.1 (3)
C23—C28—H28	120.2	C72—C71—P3	121.0 (4)
C30—C29—C34	118.0 (4)	C73—C72—C71	120.5 (4)
C30—C29—P1	122.8 (3)	C73—C72—H72	119.8
C34—C29—P1	119.1 (3)	C71—C72—H72	119.8
C31—C30—C29	120.7 (4)	C72—C73—C74	120.1 (4)
C31—C30—H30	119.7	C72—C73—H73	119.9
C29—C30—H30	119.7	C74—C73—H73	119.9
C32—C31—C30	120.6 (4)	C75—C74—C73	120.4 (4)
C32—C31—H31	119.7	C75—C74—H74	119.8
C30—C31—H31	119.7	C73—C74—H74	119.8
C31—C32—C33	119.7 (4)	C74—C75—C76	119.7 (5)
C31—C32—H32	120.1	C74—C75—H75	120.2
C33—C32—H32	120.1	C76—C75—H75	120.2
C34—C33—C32	119.7 (4)	C71—C76—C75	120.5 (4)
C34—C33—H33	120.2	C71—C76—H76	119.8
C32—C33—H33	120.2	C75—C76—H76	119.8
			
P3—Ag1—P1—C29	75.36 (15)	C26—C27—C28—C23	1.7 (7)
P4—Ag1—P1—C29	−46.32 (16)	C24—C23—C28—C27	−0.8 (6)
P2—Ag1—P1—C29	−158.28 (15)	P1—C23—C28—C27	−177.8 (3)
P3—Ag1—P1—C23	−46.74 (14)	C23—P1—C29—C30	1.2 (4)
P4—Ag1—P1—C23	−168.42 (14)	C35—P1—C29—C30	110.0 (4)
P2—Ag1—P1—C23	79.62 (14)	Ag1—P1—C29—C30	−127.0 (3)
P3—Ag1—P1—C35	−164.15 (14)	C23—P1—C29—C34	176.8 (3)
P4—Ag1—P1—C35	74.18 (15)	C35—P1—C29—C34	−74.3 (4)
P2—Ag1—P1—C35	−37.79 (15)	Ag1—P1—C29—C34	48.6 (4)
P1—Ag1—P2—C44	156.14 (18)	C34—C29—C30—C31	−0.3 (6)
P3—Ag1—P2—C44	−77.05 (18)	P1—C29—C30—C31	175.4 (3)
P4—Ag1—P2—C44	30.32 (18)	C29—C30—C31—C32	1.8 (7)
P1—Ag1—P2—C38	−79.03 (17)	C30—C31—C32—C33	−2.2 (7)
P3—Ag1—P2—C38	47.78 (17)	C31—C32—C33—C34	1.1 (7)
P4—Ag1—P2—C38	155.16 (16)	C32—C33—C34—C29	0.4 (7)
P1—Ag1—P2—C37	36.01 (14)	C30—C29—C34—C33	−0.8 (6)
P3—Ag1—P2—C37	162.82 (14)	P1—C29—C34—C33	−176.7 (4)
P4—Ag1—P2—C37	−89.80 (14)	C29—P1—C35—C36	−172.0 (3)
P1—Ag1—P3—C65	81.93 (16)	C23—P1—C35—C36	−64.8 (3)
P4—Ag1—P3—C65	−144.06 (16)	Ag1—P1—C35—C36	59.0 (3)
P2—Ag1—P3—C65	−31.36 (16)	P1—C35—C36—C37	−78.9 (4)
P1—Ag1—P3—C71	−41.11 (14)	C35—C36—C37—P2	77.1 (4)
P4—Ag1—P3—C71	92.90 (14)	C44—P2—C37—C36	173.6 (3)
P2—Ag1—P3—C71	−154.41 (13)	C38—P2—C37—C36	66.4 (3)
P1—Ag1—P3—C64	−159.07 (15)	Ag1—P2—C37—C36	−55.4 (3)
P4—Ag1—P3—C64	−25.07 (15)	C44—P2—C38—C39	145.2 (4)
P2—Ag1—P3—C64	87.63 (15)	C37—P2—C38—C39	−107.6 (4)
P1—Ag1—P4—C56	−81.76 (17)	Ag1—P2—C38—C39	11.0 (4)
P3—Ag1—P4—C56	146.44 (17)	C44—P2—C38—C43	−35.2 (5)
P2—Ag1—P4—C56	23.66 (17)	C37—P2—C38—C43	71.9 (4)
P1—Ag1—P4—C50	42.89 (17)	Ag1—P2—C38—C43	−169.4 (4)
P3—Ag1—P4—C50	−88.92 (17)	C43—C38—C39—C40	−1.0 (7)
P2—Ag1—P4—C50	148.30 (17)	P2—C38—C39—C40	178.6 (4)
P1—Ag1—P4—C62	162.07 (14)	C38—C39—C40—C41	1.9 (7)
P3—Ag1—P4—C62	30.26 (14)	C39—C40—C41—C42	−1.1 (7)
P2—Ag1—P4—C62	−92.52 (14)	C40—C41—C42—C43	−0.5 (8)
C13—Sn1—O1—C19	50.2 (3)	C41—C42—C43—C38	1.4 (9)
C7—Sn1—O1—C19	−67.4 (3)	C39—C38—C43—C42	−0.7 (8)
C1—Sn1—O1—C19	177.8 (3)	P2—C38—C43—C42	179.8 (4)
C13—Sn1—O3—C21	67.1 (3)	C38—P2—C44—C45	−71.1 (3)
C7—Sn1—O3—C21	−175.4 (3)	C37—P2—C44—C45	−178.3 (3)
C1—Sn1—O3—C21	−60.2 (3)	Ag1—P2—C44—C45	58.6 (3)
C13—Sn1—C1—C2	42.7 (4)	C38—P2—C44—C49	108.5 (3)
C7—Sn1—C1—C2	−135.7 (3)	C37—P2—C44—C49	1.3 (3)
O3—Sn1—C1—C2	137.3 (3)	Ag1—P2—C44—C49	−121.9 (3)
O1—Sn1—C1—C2	−41.7 (3)	C49—C44—C45—C46	0.1 (2)
C13—Sn1—C1—C6	−139.8 (3)	P2—C44—C45—C46	179.7 (3)
C7—Sn1—C1—C6	41.9 (4)	C44—C45—C46—C47	0.2 (3)
O3—Sn1—C1—C6	−45.1 (3)	C45—C46—C47—C48	−0.3 (5)
O1—Sn1—C1—C6	135.8 (3)	C46—C47—C48—C49	0.2 (6)
C6—C1—C2—C3	0.7 (6)	C47—C48—C49—C44	0.0 (6)
Sn1—C1—C2—C3	178.2 (3)	C45—C44—C49—C48	−0.2 (4)
C1—C2—C3—C4	−1.2 (7)	P2—C44—C49—C48	−179.7 (3)
C2—C3—C4—C5	0.4 (7)	C56—P4—C50—C55	−27.5 (4)
C3—C4—C5—C6	0.8 (7)	C62—P4—C50—C55	82.5 (4)
C4—C5—C6—C1	−1.4 (7)	Ag1—P4—C50—C55	−155.6 (3)
C2—C1—C6—C5	0.6 (6)	C56—P4—C50—C51	154.2 (3)
Sn1—C1—C6—C5	−177.0 (3)	C62—P4—C50—C51	−95.8 (4)
C13—Sn1—C7—C12	61.2 (4)	Ag1—P4—C50—C51	26.1 (4)
C1—Sn1—C7—C12	−120.2 (3)	C55—C50—C51—C52	−1.3 (6)
O3—Sn1—C7—C12	−30.8 (3)	P4—C50—C51—C52	177.1 (3)
O1—Sn1—C7—C12	152.7 (3)	C50—C51—C52—C53	0.9 (7)
C13—Sn1—C7—C8	−119.6 (3)	C51—C52—C53—C54	−0.1 (7)
C1—Sn1—C7—C8	58.9 (4)	C52—C53—C54—C55	−0.4 (7)
O3—Sn1—C7—C8	148.3 (3)	C51—C50—C55—C54	0.8 (6)
O1—Sn1—C7—C8	−28.2 (3)	P4—C50—C55—C54	−177.5 (3)
C12—C7—C8—C9	1.6 (6)	C53—C54—C55—C50	0.0 (7)
Sn1—C7—C8—C9	−177.5 (3)	C50—P4—C56—C61	112.9 (4)
C7—C8—C9—C10	−3.3 (7)	C62—P4—C56—C61	5.9 (4)
C8—C9—C10—C11	1.8 (7)	Ag1—P4—C56—C61	−111.5 (3)
C9—C10—C11—C12	1.2 (7)	C50—P4—C56—C57	−72.6 (3)
C8—C7—C12—C11	1.5 (6)	C62—P4—C56—C57	−179.7 (3)
Sn1—C7—C12—C11	−179.4 (3)	Ag1—P4—C56—C57	62.9 (3)
C10—C11—C12—C7	−2.9 (6)	C61—C56—C57—C58	−1.1 (6)
C7—Sn1—C13—C14	168.2 (3)	P4—C56—C57—C58	−175.9 (3)
C1—Sn1—C13—C14	−10.1 (4)	C56—C57—C58—C59	1.5 (7)
O3—Sn1—C13—C14	−103.6 (4)	C57—C58—C59—C60	−1.4 (7)
O1—Sn1—C13—C14	72.4 (4)	C58—C59—C60—C61	0.8 (7)
C7—Sn1—C13—C18	−8.4 (4)	C57—C56—C61—C60	0.5 (6)
C1—Sn1—C13—C18	173.3 (3)	P4—C56—C61—C60	174.9 (3)
O3—Sn1—C13—C18	79.9 (4)	C59—C60—C61—C56	−0.4 (7)
O1—Sn1—C13—C18	−104.2 (4)	C56—P4—C62—C63	−178.9 (3)
C18—C13—C14—C15	0.6 (7)	C50—P4—C62—C63	73.1 (3)
Sn1—C13—C14—C15	−176.0 (3)	Ag1—P4—C62—C63	−59.2 (3)
C13—C14—C15—C16	0.7 (7)	P4—C62—C63—C64	87.4 (4)
C14—C15—C16—C17	−1.7 (8)	C62—C63—C64—P3	−77.9 (4)
C15—C16—C17—C18	1.3 (8)	C65—P3—C64—C63	172.3 (3)
C16—C17—C18—C13	0.1 (8)	C71—P3—C64—C63	−79.7 (3)
C14—C13—C18—C17	−1.0 (7)	Ag1—P3—C64—C63	45.7 (3)
Sn1—C13—C18—C17	175.8 (4)	C71—P3—C65—C70	−159.1 (3)
Sn1—O1—C19—O2	29.7 (6)	C64—P3—C65—C70	−51.7 (4)
Sn1—O1—C19—C20	−149.2 (2)	Ag1—P3—C65—C70	71.8 (4)
O2—C19—C20—F1	6.8 (5)	C71—P3—C65—C66	26.9 (4)
O1—C19—C20—F1	−174.1 (3)	C64—P3—C65—C66	134.3 (3)
O2—C19—C20—F2	125.8 (4)	Ag1—P3—C65—C66	−102.2 (3)
O1—C19—C20—F2	−55.1 (4)	C70—C65—C66—C67	1.6 (6)
O2—C19—C20—Cl1	−113.8 (4)	P3—C65—C66—C67	175.7 (3)
O1—C19—C20—Cl1	65.3 (4)	C65—C66—C67—C68	−1.4 (7)
Sn1—O3—C21—O4	−9.4 (6)	C66—C67—C68—C69	1.1 (7)
Sn1—O3—C21—C22	171.0 (2)	C67—C68—C69—C70	−1.0 (7)
O4—C21—C22—F3	−4.1 (6)	C66—C65—C70—C69	−1.5 (7)
O3—C21—C22—F3	175.5 (3)	P3—C65—C70—C69	−175.8 (4)
O4—C21—C22—F4	−123.8 (4)	C68—C69—C70—C65	1.2 (7)
O3—C21—C22—F4	55.8 (4)	C65—P3—C71—C76	−129.0 (3)
O4—C21—C22—Cl2	115.6 (4)	C64—P3—C71—C76	123.3 (3)
O3—C21—C22—Cl2	−64.8 (4)	Ag1—P3—C71—C76	0.6 (3)
C29—P1—C23—C24	−85.8 (4)	C65—P3—C71—C72	50.4 (3)
C35—P1—C23—C24	165.2 (3)	C64—P3—C71—C72	−57.3 (4)
Ag1—P1—C23—C24	45.0 (4)	Ag1—P3—C71—C72	180.0 (3)
C29—P1—C23—C28	91.3 (4)	C76—C71—C72—C73	0.5 (6)
C35—P1—C23—C28	−17.7 (4)	P3—C71—C72—C73	−178.9 (3)
Ag1—P1—C23—C28	−137.9 (3)	C71—C72—C73—C74	−1.5 (6)
C28—C23—C24—C25	−0.5 (7)	C72—C73—C74—C75	1.3 (6)
P1—C23—C24—C25	176.7 (3)	C73—C74—C75—C76	−0.2 (6)
C23—C24—C25—C26	0.9 (7)	C72—C71—C76—C75	0.6 (6)
C24—C25—C26—C27	0.0 (7)	P3—C71—C76—C75	180.0 (3)
C25—C26—C27—C28	−1.3 (7)	C74—C75—C76—C71	−0.8 (6)

## References

[bb1] Barbour, L. J. (2001). *J. Supramol. Chem.* **1**, 189–191.

[bb2] Bruker (2007). *APEX2* and *SAINT* Bruker AXS Inc., Madison, Wisconsin, USA.

[bb3] Ng, S. W. & Hook, J. M. (1999). *Main Group Met. Chem.* **22**, 163–174.

[bb4] Sheldrick, G. M. (1996). *SADABS* University of Göttingen, Germany.

[bb5] Sheldrick, G. M. (2008). *Acta Cryst.* A**64**, 112–122. 10.1107/S0108767307043930 18156677

[bb6] Teo, Y. Y., Lo, K. M. & Ng, S. W. (2004). *Acta Cryst.* E**60**, m1478–m1480.

[bb7] Teo, Y. Y., Lo, K. M. & Ng, S. W. (2007). *Acta Cryst.* E**63**, m1365–m1367.

[bb8] Teo, Y. Y., Lo, K. M. & Ng, S. W. (2008). *Acta Cryst.* E**64**, m700. 10.1107/S1600536808010775 PMC2961194 21202233

[bb9] Tiekink, E. R. T. (1991). *Appl. Organomet. Chem.* **5**, 1–23.

[bb10] Tiekink, E. R. T. (1994). *Trends Organomet. Chem.* **1**, 71–116.

[bb11] Westrip, S. P. (2008). *publCIF* In preparation.

